# Targeting inflammation in depression: Ketamine as an anti-inflammatory antidepressant in psychiatric emergency

**DOI:** 10.1016/j.bbih.2021.100383

**Published:** 2021-11-10

**Authors:** Naghmeh Nikkheslat

**Affiliations:** Department of Psychological Medicine, Institute of Psychiatry, Psychology & Neuroscience, Kings College London, UK

**Keywords:** Major depression, Neuroinflammation, Cytokines, Ketamine, Kynurenine pathway, Anti-suicidal effect

## Abstract

Major depressive disorder (MDD) is a common psychiatric disorder with multifactorial aetiology and complex pathophysiology. Despite availability of various pharmacological and non-pharmacological therapeutic strategies, treatment resistant depression (TRD) remains a significant challenge with specific concern for those patients with severe depressive symptoms in particular suicidal ideations who require immediate and effective intervention. Inflammation has been widely studied for its association with MDD and treatment response. Ketamine known as a dissociative anaesthetic has a novel rapid-acting antidepressant effect at lower doses. Anti-inflammatory actions of ketamine appear to play a role in mechanisms underlying its antidepressant effects. Considering the rapid antidepressant action of ketamine, this review provides a brief overview of antidepressant properties of ketamine as well as its effects on peripheral and central inflammation to better understand the mechanisms underlying the therapeutic action of ketamine as an anti-inflammatory antidepressant target in psychiatric emergency. Development of effective medications, which act rapidly with dual effect on both inflammation and MDD would be of a significant clinical importance for a successful and personalised treatment of inflammatory-induced TRD and suicidal thoughts and behaviour.

## Highlights

• Anti-inflammatory actions of ketamine play a role in mechanisms underlying its antidepressant effects.
• Ketamine’s dual effect on inflammation and depression is particularly important in treatment of inflammatory-induced TRD and suicidal patients.
• Ketamine affects CNS receptors and pathways, neurotransmitter systems, synaptogenesis, and inflammatory responses.
• Ketamine remains a promising target for treatment of TRD and suicidal thoughts.

## 1 Introduction

As one of the most common psychiatric and mental illnesses, major depressive disorder (MDD) has been investigated extensively for decades, and yet the complex multifactorial aetiology and mechanisms underlying its pathophysiology need to be understood. Despite availability of various pharmacological and non-pharmacological therapeutic strategies, treatment resistant depression (TRD) remains a significant challenge with specific concern for those patients who present severe depressive symptoms and particularly suicidal ideations and behaviours (Bergfeld et al., 2018; Orsolini et al., 2020; Voineskos et al., 2020).

**Fig. 1:**
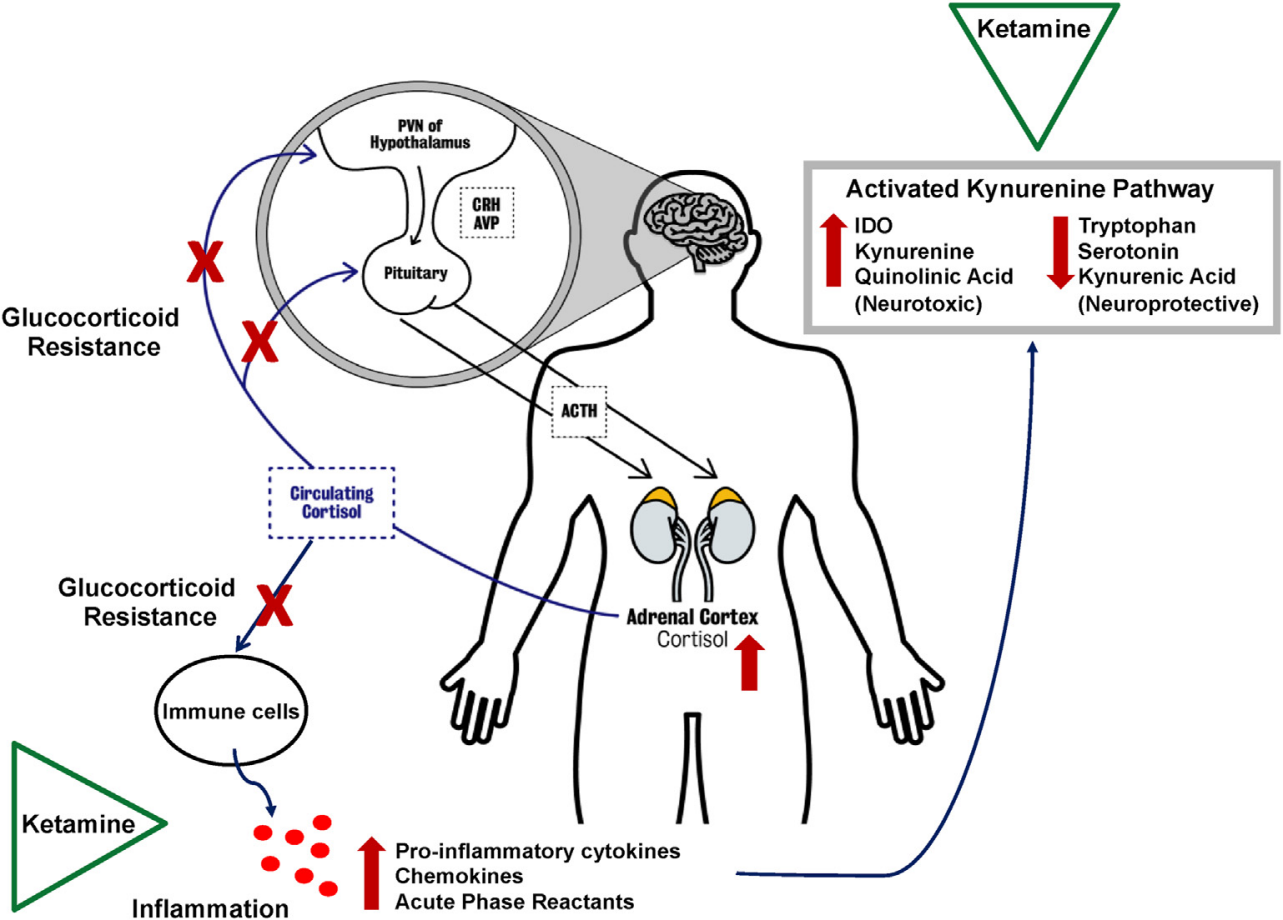
**Dysregulation of HPA axis activity in depression and the putative targets of ketamine anti-inflammatory actions -** Hyperactivation of the hypothalamic-pituitary-adrenal (HPA) axis, glucocorticoid resistance, immune cells alteration, excessive release of inflammatory biomarkers, and activated kynurenine pathway are implicated in pathophysiology of depression. Dysregulation of the HPA axis seems to reflect an alteration in glucocorticoid receptors functional properties and an impaired ability of cortisol to exert its physiological effects (glucocorticoid resistance) including the negative feedback on the HPA axis itself as well as the anti-inflammatory effects on the immune cells and release of inflammatory biomarkers. Inflammatory mediated activation of the kynurenine pathway results in less availability of tryptophan and reduced serotonin biosynthesis. Inflammatory induced IDO activation leads to an increase in the production of kynurenine and quinolinic acid (neurotoxic metabolite) and a decrease in kynurenine acid (neuroprotective metabolite). Ketamine exerts its anti-inflammatory actions by directly affecting immune cells and inhibiting the production and release of inflammatory biomarkers including pro-inflammatory cytokines as well as modulating cytokine-induced activation of kynurenine pathway and decreasing neurotoxic quinolinic acid thus attenuating neuroinflammation and neurodegeneration. AVP= arginine vasopressin; ACTH= adrenocorticotrophic hormone; CRH= corticotropic releasing hormone; IDO= indoleamine-2,3-dioxygenase.

Advances in psychoneuroimmunology research led to growing reports on the involvement of inflammation in pathophysiology of MDD. Studies from our laboratory and others have been consistently shown that alterations in the function of hypothalamic-pituitary-adrenal (HPA) axis and ineffective glucocorticoid signalling leads to inappropriate immune and inflammatory responses in MDD (Fig. 1). Abnormal communication between the periphery and the central nervous system (CNS) provokes neuroinflammation, which further induces glucocorticoid resistance within the brain. Activated inflammatory mediators induce depressive symptoms through direct effect on the brain tissue, modulation of the serotonergic system and initiation of neurodegenerative processes. Inflammatory induced activation of the kynurenine pathway results in less availability of tryptophan for the serotonin biosynthesis, and instead a shift towards production of kynurenine and downstream neurotoxic metabolites, and ultimately neurodegeneration (Nikkheslat et al., 2015, 2018; Sforzini et al., 2019). The association between inflammation and depression appears to be even independent of genetic, health, and psychosocial factors (Pitharouli et al., 2021). Evidence suggests presence of activated immune response and increased immune cells such as neutrophils and monocytes (Lynall et al., 2020) as well as elevated levels of inflammatory biomarkers such as cytokines and C-reactive protein (CRP) in a significant proportion of depressed patients (Nikkheslat et al., 2018); however, the phenomenon is found to be more prevalent in those less responsive to antidepressant treatments (Cattaneo et al., 2020; Haroon et al., 2018; Nikkheslat et al., 2019; Yang et al., 2019) and with suicidal thoughts and attempts (Serafini et al., 2013). Effective antidepressant medications have been shown to restore the neuro-endocrine-immune balance by normalising the HPA axis dysregulation (Himmerich et al., 2006; Horowitz et al., 2014; Nikkheslat et al., 2017).

Ketamine is approved as a safe and effective dissociative anaesthetic since 1960s (Dundee et al., 1970) and at lower doses is a promising novel treatment as a rapid-acting antidepressant (Zanos et al., 2018). The effects on core features of depression are detectable within a few hours (Gould et al., 2019). This potential rapid effect is quite a unique feature, in comparison to classical antidepressants, which take at least two weeks to exert their therapeutic effects that in severe cases could leave a life-threatening situation for the patients. While the mechanisms behind the antidepressant effects of ketamine are not fully understood, anti-inflammatory effects are candidates. Anaesthetics have been studied for their immunosuppressive properties in relation to clinical implications of the use of these agents during surgical procedures. While different anaesthetics affect the immune system differentially, they seem to either act on the HPA axis and affect its immunomodulatory activity or directly modulate the function of immune cells and depress inflammatory responses (Cruz et al., 2017). The anti-inflammatory effect of ketamine has been investigated in clinical and preclinical studies and will be discussed in the present review.

There are increasing numbers of clinical trials conducted by our clinical research team and others, on improving the treatment response in MDD, which have been trying to address the inflammation through the potential therapeutic benefit of using a combination of anti-inflammatory and antidepressant medications (Fourrier et al., 2018; Miller and Pariante, 2020; Nettis et al., 2021). While there is no question about translational and clinical significance of these studies in treatment of MDD and TRD when there is a link to inflammation, the approach still requires the time frame needed for the drug's effects, thus the urgent need for development of alternative treatment strategies for those patients with severe depressive symptoms in particular suicidal ideations who require effective but immediate intervention (Gladwell, 2021). Therefore, considering the rapid antidepressant action of ketamine, this review provides a brief overview of antidepressant properties of ketamine as well as its effects on peripheral and central inflammation to better understand the mechanisms behind the therapeutic action of ketamine as an anti-inflammatory antidepressant target. Development of effective medications, which act rapidly with dual effect on both inflammation and depression would be of a significant clinical importance, especially for those depressed individuals who are at an imminent risk of suicide and treatment resistant due to the presence of inflammation.

## 2 Antidepressant property of ketamine

Traditionally used as an anaesthetic, ketamine was first claimed to have an antidepressant effect in 2000 as reported by a placebo-controlled trial showing that depressed patients evidenced significant alleviation in depressive symptoms within 72 ​h following a single low-dose ketamine infusion (Berman et al., 2000). Another randomized controlled trial investigated the efficacy of ketamine in TRD and found a robust and rapid antidepressant response, which manifested within 2 ​h, peaked at around 24 ​h and remained relatively sustained in 35% of patients for 1 week (Zarate et al., 2006). The findings on effectiveness of a single-dose intravenous ketamine in rapidly improving depressive symptoms in TRD were further replicated by several clinical trials and confirmed by meta-analysis (Kishimoto et al., 2016). Repeated ketamine infusions were also studied in patients with TRD revealing cumulative and sustained antidepressant effects in responders with no reported serious side effects (Phillips et al., 2019). Ketamine as a rapid-acting antidepressant is an attractive candidate for psychiatric emergency, and its effectiveness has been studied in TRD patients with suicidal ideation. Initial studies showed a single-intravenous infusion of ketamine reduced suicidal thoughts within 40 ​min and the effect remained for up to 4 ​h post-infusion (DiazGranados et al., 2010; Thakurta et al., 2012) and sustained for 12 days by repeated-dose ketamine administration (Price et al., 2009). A report from a meta-analysis examining the effects of a single-dose ketamine on suicidal ideation in MDD confirmed rapid reduction in suicidal thoughts in patients within one day and for up to one week in response to ketamine's effects, which were found to be partially independent of the antidepressant effects, suggesting involvement of specific mode of actions (Wilkinson et al., 2018). A recent randomized controlled trial assessing the effect of single and repeated ketamine infusions on suicidal ideation in TRD patients showed rapid and cumulative reduction in suicidal thoughts with thrice-weekly repeated infusions that was prolonged with once-weekly maintenance treatment in responders (Phillips et al., 2020).

## 3 Mechanisms of action

Ketamine is a non-competitive inhibitor of glutamate N-methyl-d-aspartate (NMDA) receptor, and this antagonism property is likely to play a central role to its mechanism of action as an antidepressant. Dysfunction of glutamatergic system including abnormalities in glutamate and the NMDA receptor, is involved in pathophysiology of MDD and TRD and psychopathology underlying suicide (Deutschenbaur et al., 2016; Sanacora et al., 2012). Ketamine exerts antidepressant effects by blocking NMDA receptors located on inhibitory ^γ^-aminobutyric acid (GABA) interneurons, thus preventing activation of GABA neurons which results in disinhibition of glutamate transmission. Excessive extracellular glutamate activates synaptic α-amino-3-hydroxy-5-methyl-4-isoxazolepropionic acid (AMPA) receptors which in turn increases brain-derived neurotrophic factor (BDNF) release promoting synthesis of synaptic proteins, formation of dendritic spines and synapses strength. Indeed, synaptogenesis is potentiated by mechanisms involving the BDNF-mediated stimulation of the tropomyosin receptor kinase B (TrkB) receptor and subsequent activation of mechanistic target of rapamycin (mTOR) signalling pathway (Matveychuk et al., 2020). Pre-clinical studies have shown that the effects of ketamine on synapse formation and antidepressant-like behaviour are mTOR dependent (Yang et al., 2013; Zhou et al., 2014) and that these effects are reversed by pre-treatment with the selective mTOR inhibitor rapamycin infusion and blocking the mTOR signalling (Li et al., 2010). Interestingly, rapamycin pre-treatment in MDD patients did not reduce the rapid antidepressant effects of ketamine but increased the response and remission rates at 2 weeks that may highlight the need for further investigation on the role of systemic versus local blockade of mTOR pathway in association with ketamine's antidepressant actions (Abdallah et al., 2020). Ketamine-mediated antagonism of NMDA receptors also directly acts on BDNF pathway resulting in de-suppression of BDNF translation via deactivation of eukaryotic elongation factor 2 kinase, thus reversing deficits in stress-induced synaptic plasticity (Deutschenbaur et al., 2016). The mechanisms of ketamine action are not limited to its high affinity to NMDA receptors and the effects on AMPA and GABA receptors, but also its interaction with other major neurotransmitter systems, such as dopaminergic, serotoninergic, adrenergic, and cholinergic pathways (Zanos et al., 2018) that are implicated in mood disorders.

Used as an anti-nociceptive agent for neuropathic chronic pain (Niesters et al., 2014), ketamine activates opioid system, the mechanism which is suggested to be necessary for ketamine's rapid antidepressant effects requiring both NMDA and opioid receptor signalling and interactions between these two neurotransmitter systems (Klein et al., 2020). Indeed, pre-treatment with opioid-receptor antagonists has been shown to attenuate some antidepressant effects of ketamine (Williams et al., 2018). The anti-suicidal effects of ketamine have been also linked to the ketamine-induced activation of opioid receptor (Williams et al., 2019). However, ketamine's anti-suicidal response appears not to be completely driven by its antidepressant's effects (Ballard et al., 2014) as evidenced by studies finding an association of reduced suicidal ideation with increased regional cerebral glucose metabolism (Ballard et al., 2015) and decreased night-time wakefulness following ketamine infusion (Vande Voort et al., 2017), which were not associated with improved depressive symptoms. Ketamine-induced plasticity in relation to modulation of BDNF pathway and improvement in sleep pattern may explain neurobiological mechanism underlying the anti-suicidal effects (Duncan et al., 2013).

Ketamine metabolizes rapidly within minutes and is distributed quickly in highly perfused tissues. The rapid transfer of ketamine across the blood-brain-barrier into the brain is facilitated by its liposolubility and low plasma protein binding properties (Haas and Harper, 1992). Structurally, ketamine is a racemic mixture of R- and S-ketamine and the effect of ketamine is suggested to be through the function of its active metabolites most notably hydroxynorketamines. The versatile mode of action of ketamine targeting various systems in a distinct but complementary manner as well as its structure and metabolites are believed to underlie its unique therapeutic effect (Sial et al., 2020). S-ketamine known as Esketamine is more potent NMDA receptor antagonist than R-ketamine and has been approved by the Food and Drug Administration (FDA) in 2018 for treatment of TRD (Kim et al., 2019) and in 2020 for treatment of MDD with acute suicidal ideation or behaviour (Mischel and Balon, 2021). Randomised controlled trials reported rapid onset of antidepressant effects in patients with MDD within 2 ​h of intravenous esketamine infusion (Singh et al., 2016), and 24 ​h after intranasal esketamine given in addition to oral antidepressant therapy (Daly et al., 2018) with a significant effect in delaying relapses when used long-term for up to 16 weeks (Daly et al., 2019) and a sustained effect and manageable safety profile when used for up to 48 weeks in responders (Wajs et al., 2020). The effectiveness of esketamine nasal spray is also observed in severe cases of MDD with active suicidal ideation with intent (Canuso et al., 2018; Ionescu et al., 2021).

## 4 Adverse effects

Beside the unique therapeutic potential, ketamine elicits acute but transient adverse effects including dissociative symptoms, blood pressure elevation, tachycardia, urologic toxicity, and perceptual disturbances. Even though many clinical studies confirmed the efficacy and safety of ketamine, its regular use as an antidepressant is restricted due to the psychotomimetic effects, drug abuse potential and dissociative properties (Matveychuk et al., 2020). A recent meta-analysis of placebo-controlled crossover ketamine trials comprehensively assessed both dissociative and non-dissociative side effects associated with a single intravenous subanaesthetic dose of ketamine in TRD and reports that most symptoms peaked within 1 ​h, resolved by 2 ​h, and none lasted for more than 4 ​h. The most common identified side effects were feeling strange, weird, and loopy. No serious long-lasting adverse effects such as cystitis, anaphylaxis, emergence delirium, cognitive or memory deficits, increased tendency for ketamine use or abuse were observed after 3 months follow-up assessments (Acevedo-Diaz et al., 2020). However, the evaluation of long-term and routine application of ketamine in treatment of MDD in large-scale clinical trials merits further investigations (Short et al., 2018).

While ketamine has a rapid-acting property, its short effect duration and the requirements for repeated administrations and maintenance regimen are not desirable specially when applied via intravenous injections which requires hospital or clinic setting (Pribish et al., 2020). Although commonly employed via intravenous infusion for obtaining the highest drug bioavailability, other more practical routes of administrations including oral, sublingual, and intranasal can be considered at least after initial dosing (Andrade, 2017). however, the overall side effect profile limits the usage of ketamine outside clinical environment and requires a risk assessment for each patient (Schoevers et al., 2016). Adhering to good clinical management remains essential for achieving an adequate therapeutic response and sustaining long term effects. According to recommended guidelines, an effective treatment strategy would consider continued treatment with conventional antidepressants and adjunctive psychotherapeutic interventions (Kennedy et al., 2016; Talbot et al., 2019; Wilkinson et al., 2017).

Evidence from animal studies encourages clinical investigation into comparing the antidepressant effects and adverse effects of the ketamine stereoisomers. R-ketamine was found to produce rapid and long-lasting effects on depression-like behaviour in juvenile mice after neonatal dexamethasone exposure, relative to S-ketamine (Zhang et al., 2014). In addition, in animal models, R-ketamine appeared to exhibit a better side effect profile than S-ketamine and was shown to be a more efficacious and safer antidepressant, free of psychotomimetic side effects and abuse liability (Yang et al., 2015a). The more sustained antidepressant effect of R-ketamine was also confirmed in a treatment refractory model that was found to be mediated through AMPA receptor stimulation (Fukumoto et al., 2017). While the approval and practise of esketamine in MDD is still an active and ongoing topic in scientific discussions (Mischel and Balon, 2021), the investigations into superior potency of R-ketamine's effects over S-ketamine has not yet been reported in clinical settings. Better understanding of the wide range of mechanisms through which ketamine exerts its ultra-rapid distinctive therapeutic actions remains essential for development of effective, safe, and personalised treatment strategies.

## 5 Anti-inflammatory property of ketamine

The effect of ketamine on inflammation has been of particular interest since the drug has been used as an anaesthetic in patients undergoing surgery and has been found to be acting as a unique homeostatic regulator of the stress-induced immune disturbances and the acute inflammatory reactions (De Kock et al., 2013). Regulation of inflammatory responses is considered as a vital contributing factor in surgery outcome and recovery. Evidence from clinical studies show intraoperative ketamine administration attenuates inflammatory reactivity following major surgeries including cardiac and abdominal operations as observed by significant inhibition of the early postoperative interleukin (IL)-6 inflammatory response (Dale et al., 2012). In obese patients, ketamine attenuated production of IL-6 and preserved immune responses as measured by lymphocyte proliferation and natural killer cell cytotoxicity after a short-duration surgery (Roussabrov et al., 2008). Even subanaesthetic dose of ketamine prior to induction of general anaesthesia was shown to result in modulation of immune cells in the early postoperative period as observed by *ex vivo* attenuation of IL-6 and tumour necrosis factor (TNF)-α production and preservation of IL-2 secretion (Beilin et al., 2007). The anti-inflammatory property of ketamine is considered significant due to its effect on limiting and even preventing exaggerated systemic inflammation without interfering with local essential healing processes. Ketamine appears to exert anti-inflammatory activity in the context of an increased immune activation, acting as an anti-pro-inflammatory agent rather than an immunosuppressant (Loix et al., 2011). This immunomodulatory function in combination with antidepressant property make ketamine a highly desirable candidate for treatment of subgroups of MDD patients who present elevated inflammation.

The past decade has seen an emergence of research on the immunomodulatory property of ketamine in MDD and the regulation of inflammation as a mechanism underlying its rapid antidepressant effects. Ketamine has been shown to have a direct effect on peripheral leucocytes and suppresses the proinflammatory cytokine production (Kawasaki et al., 1999). The authors demonstrated significant inhibition of lipopolysaccharide (LPS)-induced TNF-α, IL-6, and IL-8 production in human whole blood. The anti-inflammatory actions, which contributes to ketamine's antidepressant effects is also evidenced by other *in vitro* studies reporting that ketamine inhibits the production and release of pro-inflammatory cytokines, IL-1β, IL-6 and TNF-α in macrophages (Chang et al., 2010), microglial cells (Chang et al., 2009), and astrocytes (Yuhas et al., 2015). The association between ketamine rapid antidepressant property and its anti-inflammatory effect is further supported by animal studies. Ketamine has been shown to alleviate stress-induced depressive-like behaviours as studied in a chronic unpredictable mild stress model of depression with up-regulated levels of IL-1β, IL-6, and TNF-α cytokines; and that the effects on the measures of anhedonia, behavioural despair, and neurovegetative changes were associated with down-regulation of the hippocampal inflammatory response (Wang et al., 2015). The mechanism through which ketamine exerts its anti-inflammatory antidepressant effect in mice was that ketamine decreased the number of activated microglia cells in the hippocampus, reduced the levels of IL-1β, IL-6, and TNF-α, down-regulated cytokine synthesis through the TLR4/p38 signalling pathway, and inhibited cytokine release from microglia by down-regulating P2X7 receptor in hippocampus (Tan et al., 2017).

Even though there are still limited data available from clinical studies and some even reported contradictory findings (Park et al., 2017), ketamine has been found to exert antidepressant effects mediated by its anti-inflammatory actions. Adipokines which are compounds involved in regulation of inflammation and neuroplasticity pathways (Machado-Vieira et al., 2017), and serum inflammatory marker IL-6 (Yang et al., 2015b) have been shown to predict ketamine's antidepressant response in TRD. In a recent randomised controlled trial, the rapid improvement in depressive symptoms in patients with TRD is shown to be related to the rapid suppression of TNF-α. In addition, it appears that ketamine exerts its anti-inflammatory effects at higher dose of 0.5 ​mg/kg compared to dose of 0.2 ​mg/kg by which the antidepressant effects are present suggesting involvement of other mechanisms at lower doses (Chen et al., 2018). In accordance with pre-clinical studies, which provide strong support for ketamine-induced decreases in pro-inflammatory cytokines, overall clinical evidence also demonstrates reduction of peripheral inflammatory markers including IL-1β, IL-6, and TNF-α, as reported by our recent systematic review (Kopra et al., 2021).

The central immunomodulatory effect of Ketamine is evident by its direct effect on glial cells (Zhang et al., 2021). The association of microglial activation and release of cytokine TNF-α and nitric oxide, which are key mediators of acute and chronic inflammatory and neurodegenerative processes, have been found in depressed patients with suicide ideation (Steiner et al., 2008). Microglial cells can also affect regulation of BDNF synthesis and reduce BDNF expression and its high-affinity receptor TrkB (Jin et al., 2019). A post-mortem study reported elevation of primed phenotype of microglial and accumulation of cerebral macrophages in the brain of depressed suicides (Torres-Platas et al., 2014). Ketamine has been found to inhibit LPS-stimulated production of inflammatory response TNF-α in both astrocytes and microglia (Shibakawa et al., 2005). Ketamine effect on microglial inactivation appears to be mediated by inhibition of extracellular signal-regulated kinase phosphorylation as studied in primary cultures from rats *in vitro* (Chang et al., 2009). Using HMC3 human microglial cell line, it has been shown that ketamine and its active metabolites are involved in regulation of the type I interferon pathway mediated through signal transducer and activation of transcription 3, which plays a crucial role in the immune response, as well as augmentation of BDNF expression (Ho et al., 2019). In cultured human astroglial cells, ketamine supressed gene expression and production of IL-6 and TNF-α within 24 ​h, that further supports the link between the ketamine immunomodulatory activity and its rapid antidepressant effect (Yuhas et al., 2015).

Ketamine action through an involvement of kynurenine pathway explains another anti-inflammatory mechanism through which ketamine may exert its antidepressant effect (Zunszain et al., 2013). Cytokine-induced activation of kynurenine pathway of tryptophan metabolism and an imbalance between neurotoxic and neuroprotective metabolites is implicated in MDD through the effects on glutamatergic neurotransmission (Savitz, 2017; Ogyu et al., 2018). Glutamatergic system has been widely linked, through neurotoxicity, with both neuroinflammation and depression (Cui et al., 2019). Studies on MDD patients with suicidal ideation has shown rapid elevation of NMDA receptor antagonist kynurenic acid (kynurenine neuroprotective metabolite) and higher kynurenic acid/kynurenine ratio, which were associated with reduction of depressive symptoms in ketamine responders (Zhou et al., 2018). Compared to healthy controls, suicide attempters show decreased cerebrospinal fluid (CSF) kynurenic acid and increased NMDA receptor agonist quinolinic acid (kynurenine neurotoxic metabolite), which is produced by inflammatory-induced activation of indoleamine 2,3-dioxygenase enzyme, which diverts tryptophan degradation into kynurenine and downstream neurotoxic pathway (Bay-Richter et al., 2015). Ketamine has been also found to have a direct effect on quinolinic acid by blocking its impact on the NMDA receptor as evidenced by pre-clinical studies (Walker et al., 2013) and has been suggested to benefit depressed patients with elevated inflammation before other anti-inflammatory treatment are used as maintenance strategies (Miller 2013). The direct effect of ketamine on quinolinic acid is important due to neuroactivity of this metabolite involving overstimulation of NMDA receptors, oxidative stress, neuroinflammation, and apoptosis which ultimately leads to neurodegeneration (Lugo-Huitrón et al., 2013). Considering the presence of low-grade inflammation and increased levels of quinolinic acid in the CSF of suicide attempters (Erhardt et al., 2013), the elegant anti-inflammatory property of ketamine appears to be directly relevant to its anti-suicidal effect.

## 6 Conclusion ad future directions

Beside holding a novel mechanism of action that is distinct from conventional antidepressant drugs, having also anti-inflammatory property makes ketamine particularly unique for targeting inflammatory-induced complications in depression. Diverse and multifunctional properties of ketamine including the effects on CNS receptors and pathways, neurotransmitter systems, synaptogenesis, modulation of central and peripheral inflammatory responses, which are all mechanisms underlying its antidepressant and anti-inflammatory actions, implicate ketamine in management of inflammation in MDD (Fig. 1). However, greater clinical studies in required to validate the stronger and more consistent evidence from preclinical models on the association between anti-inflammatory and antidepressant effects of ketamine as well as to compare the effect of different ketamine stereoisomers. Future studies are warranted to investigate R-ketamine in treatment of TRD and suicidal ideation and in relation to inflammation. In addition, comprehensive assessment of inflammatory profile by including broad range of peripheral inflammatory and HPA axis biomarkers as well as complete evaluation of kynurenine pathway metabolites would provide wider picture for addressing the dual effect of ketamine in both inflammation and depression. Ketamine remains a promising target for treatment of TRD and suicidal thoughts, and rapid changes in inflammatory markers should be studied as potential mediators of these therapeutic effects, especially in subset of patients with higher levels of inflammation. Further investigations of the pathways underlying the effects of ketamine on inflammation in depression is crucial for enhancing our understanding of the therapeutic efficacy of this medication as an anti-inflammatory antidepressant and provides mechanistic insight for developing next generation of rapid-acting antidepressant agents in order to achieve successful clinical treatment of inflammatory-induced TRD and suicidal ideation and behaviour.

## Declaration of conflicting interest

The author confirms no potential conflicts of interest for this review.
